# Alternative Oxidase: A Mitochondrial Respiratory Pathway to Maintain Metabolic and Signaling Homeostasis during Abiotic and Biotic Stress in Plants

**DOI:** 10.3390/ijms14046805

**Published:** 2013-03-26

**Authors:** Greg C. Vanlerberghe

**Affiliations:** Department of Biological Sciences and Department of Cell and Systems Biology, University of Toronto Scarborough, 1265 Military Trail, Toronto, ON, M1C1A4, Canada; E-Mail: gregv@utsc.utoronto.ca; Tel.: +1-416-208-2742; Fax: +1-416-287-7676

**Keywords:** alternative oxidase, carbon and energy metabolism, metabolic homeostasis, plant mitochondria, photosynthesis, reactive nitrogen species, reactive oxygen species, respiration, abiotic and biotic stress, signaling homeostasis

## Abstract

Alternative oxidase (AOX) is a non-energy conserving terminal oxidase in the plant mitochondrial electron transport chain. While respiratory carbon oxidation pathways, electron transport, and ATP turnover are tightly coupled processes, AOX provides a means to relax this coupling, thus providing a degree of *metabolic homeostasis* to carbon and energy metabolism. Beside their role in primary metabolism, plant mitochondria also act as “signaling organelles”, able to influence processes such as nuclear gene expression. AOX activity can control the level of potential mitochondrial signaling molecules such as superoxide, nitric oxide and important redox couples. In this way, AOX also provides a degree of *signaling homeostasis* to the organelle. Evidence suggests that AOX function in metabolic and signaling homeostasis is particularly important during stress. These include abiotic stresses such as low temperature, drought, and nutrient deficiency, as well as biotic stresses such as bacterial infection. This review provides an introduction to the genetic and biochemical control of AOX respiration, as well as providing generalized examples of how AOX activity can provide metabolic and signaling homeostasis. This review also examines abiotic and biotic stresses in which AOX respiration has been critically evaluated, and considers the overall role of AOX in growth and stress tolerance.

## 1. Introduction

### 1.1. Plant Respiration and the Plant Mitochondrial Electron Transport Chain

Photosynthesis and respiration are the primary pathways of carbon and energy metabolism in plants. Photosynthesis uses light energy, CO_2_ and H_2_O to drive the synthesis of carbohydrates and release of O_2_. Respiration then uses these carbohydrates to support growth and maintenance through the provision of carbon intermediates, reducing equivalents and ATP. These processes, in turn, release CO_2_ and convert O_2_ back to H_2_O.

**Figure 1:**
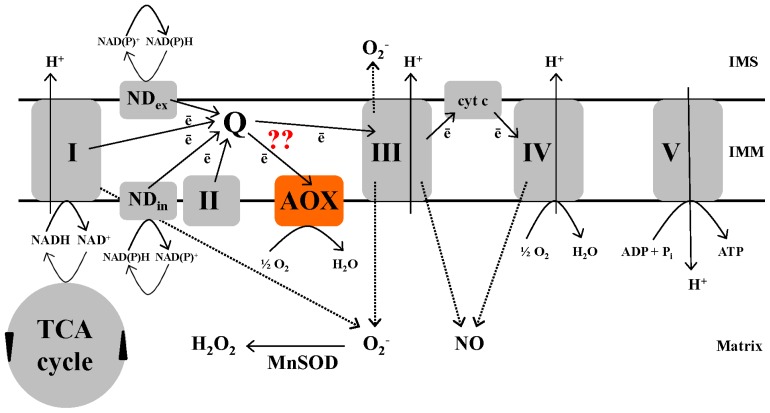
The plant mitochondrial electron transport chain. NADH oxidation by complex I is coupled to proton transport from the matrix to IMS, while NAD(P)H oxidation by a series of alternate dehydrogenases is not coupled to proton transport. Similarly, electron flow from ubiquinol to complex IV (reducing O_2_ to H_2_O) is coupled to proton transport (at two sites) while electron flow from ubiquinol to AOX (also reducing O_2_ to H_2_O) is not coupled to proton transport. Proton transport generates a proton motive force that is subsequently dissipated by ATP synthase (complex V) to produce ATP. Plants can therefore modulate their ATP yield depending on the components of the ETC being used for NAD(P)H oxidation and O_2_ reduction. When the ability of an ETC component to transport electrons is reduced and/or membrane potential is high, electron transport can slow, leading to an over-reduction of the ETC. Under these conditions, single electron leak to O_2_ or nitrite increases, producing O_2_^−^ and NO, respectively. In plants, the specific sites and mechanisms of O_2_^−^ and NO generation are not yet well understood. See text for further details. I, II, III, IV, V: complexes I to V, IMS, inner membrane space; IMM, inner mitochondrial membrane; MnSOD, manganese superoxide dismutase; ND_in_, internal-oriented alternate NAD(P)H dehydrogenases; ND_ex_, external-oriented alternate NAD(P)H dehydrogenases; Q, ubiquinone pool.

Glycolysis, the oxidative pentose phosphate (OPP) pathway and the mitochondrial tricarboxylic acid (TCA) cycle are the central respiratory pathways using photosynthesis-derived carbohydrate to supply carbon intermediates for biosynthesis, as well as coupling carbon oxidation with the reduction of NAD(P) to NAD(P)H. These reducing equivalents are then used to support biosynthetic reactions or can be oxidized by the mitochondrial electron transport chain (ETC), localized in the inner mitochondrial membrane (IMM). Complex I (NADH dehydrogenase), as well as a series of other plant-specific “alternate” dehydrogenases couple NAD(P)H oxidation to reduction of the ubiquinone pool. Complex II (succinate dehydrogenase of the TCA cycle) is a further supply of electrons for the ubiquinone pool. Electrons in ubiquinol are then passed to complex III, cytochrome (cyt) c and finally complex IV (cyt oxidase), which catalyzes the four-electron reduction of O_2_ to H_2_O. Importantly, electron transport at complexes I, III and IV is coupled with proton translocation from the mitochondrial matrix to inner membrane space (IMS) and the resulting proton motive force is used by complex V (ATP synthase) to generate ATP from ADP and P_i_[1] (Figure 1).

A defining feature of the plant mitochondrial ETC is the presence of two terminal oxidases (Figure 1). In addition to cyt oxidase, an alternative oxidase (AOX) is present that directly couples the oxidation of ubiquinol with the reduction of O_2_ to H_2_O. AOX introduces a branch in the ETC, such that electrons in ubiquinol are partitioned between the cyt pathway (complex III, cyt c, complex IV) and AOX. Notably, AOX dramatically reduces the energy (ATP) yield of respiration since it is not proton pumping and since electrons flowing to AOX bypass the proton pumping complexes III and IV. Electron flow to AOX can still support a reduced ATP yield if these electrons arise via the proton-pumping complex I. However, if electron flow to AOX is being supported by an alternate dehydrogenase or by complex II (that, unlike complex I, are not proton-pumping), then electron flow will be completely uncoupled from ATP turnover. In summary, plants have additional ETC components that allow for a dramatic modulation of ATP yield depending on the components of the path used for NAD(P)H oxidation and O_2_ reduction [1,2] (Figure 1).

### 1.2. Respiratory Metabolism and Plant Stress Biology

As sessile organisms, land plants are subjected to many stressors in their environment such as high or low temperature, drought, nutrient deficiency, salt and metal toxicity, hypoxia, and pathogen attack. Since the net carbon gain of a plant is equal to CO_2_ uptake by photosynthesis minus CO_2_ release by respiration, changes in either of these processes during stress will impact overall plant growth and productivity [3–5].

Considerable understanding has been gained regarding the impact of different stresses on photosynthetic metabolism [6,7]. Much less has traditionally been known about the impact of stress on respiration, but a surge of interest in recent years is beginning to provide important insight. For example, the glucose-6-phosphate dehydrogenase (G6PDH) step in the cytosolic OPP pathway generates NADPH that is critical for the establishment of stress tolerance. On the one hand, it was shown that G6PDH was key to providing sufficient substrate for the plasma membrane NADPH oxidase that, in response to biotic stress, generates a reactive oxygen species (ROS) “signal” important to initiate stress acclimation strategies, such as the hypersensitive response (HR) [8]. On the other hand, activation of G6PDH during salt stress was critical to supporting ROS-scavenging systems of the cell (which use NADPH as electron source) to prevent oxidative damage [9]. These examples illustrate that a single metabolic enzyme can have complex and varied roles in stress tolerance. They also emphasize that ROS are a common theme in plant stress biology, conversely acting as either damaging agents to be avoided or as signaling molecules important for stress acclimation responses.

### 1.3. Mitochondrial Reactive Oxygen and Reactive Nitrogen Species

Plant mitochondria are a source of ROS (Figure 1). This is primarily due to “single electron leak” from respiratory chain components to O_2_ producing superoxide (O_2_^−^). Both complexes I and III are proposed to be major sites of such electron leak [10,11]. Both complexes release O_2_^−^ to the matrix, while complex III may also release some O_2_^−^ to the IMS, as shown in animals. Once produced, matrix O_2_^−^ can be further converted to H_2_O_2_ by a matrix-localized manganese superoxide dismutase (MnSOD) [12]. The generation of reactive nitrogen species (RNS) such as nitric oxide (NO) has also been linked to plant mitochondria. However, the mechanism and site(s) of generation of RNS by mitochondria is not yet well understood [13,14]. In recent years, there is growing evidence that ROS and RNS specifically generated by mitochondria influence plant responses to stress, suggesting that they act as signaling molecules for stress acclimation [15,16].

### 1.4. The Plant Mitochondrial Stress Response

Mitochondria are the hub of respiratory metabolism. Hence, it is important to determine the impact of abiotic and biotic stress on the function of this organelle, and to understand means by which the cell acclimates to preserve mitochondrial function under such conditions. Significant progress has been made in recent years to establish the response of the plant cell to specific mitochondrial dysfunctions. In part, such studies have been aimed to characterize the retrograde response, whereby the functional status of the mitochondrion controls the expression of nuclear genes encoding mitochondrial proteins [17]. For example, microarray analyses have documented the transcriptional responses to complex I dysfunction induced by the inhibitor rotenone [18], complex III dysfunction induced by the inhibitor antimycin A (AA) [19,20], aconitase dysfunction induced by monofluoroacetate [20] and ATP synthase dysfunction due to mutation [21,22]. A common theme emerging from these and other studies is that the transcriptional responses have significant overlap with responses to abiotic and biotic stress [20,23–25]. This suggests that mitochondrial function is being significantly compromised during stress, hence evoking a “mitochondrial stress response”. Many of the above studies have also characterized proteomic and/or metabolic changes in response to specific mitochondrial perturbations, as have studies of other specific mitochondrial perturbations [12,26–38]. Collectively, this growing body of literature provides an important start point to comprehensively establish how mitochondria are impacted by stress and what acclimation strategies are employed by the cell.

A notable feature of the above studies is that diverse mitochondrial dysfunctions, often associated with oxidative stress, results in the induction of AOX at the transcript and protein level. As a result, AOX is now often used as a general marker of mitochondrial dysfunction and/or cellular oxidative stress. Further, numerous abiotic and biotic stress conditions are known to elevate AOX amount, supporting the idea that such stresses impact mitochondrial function and that AOX might represent an important acclimation response. The purpose of this review article is to discuss the roles(s) of AOX during plant stress. Section 2 will provide background to AOX respiration, including an introduction to known genetic and biochemical controls. Section 3 will outline means by which AOX respiration could be acting to support homeostasis in carbon and energy metabolism. Similarly, Section 4 will outline how AOX could provide a degree of homeostasis to potential signaling functions of the mitochondrion. Section 5 will then highlight studies of AOX respiration during different abiotic and biotic stress, emphasizing how these studies might relate to providing metabolic and/or signaling homeostasis under stress. Section 6 will review studies relevant to assessing the importance of AOX in plant performance and plant tolerance of different stress conditions. Finally, Section 7 provides a brief conclusion.

## 2. Alternative Oxidase

### 2.1. Brief Background

Genes encoding AOX are ubiquitous in the Kingdom Plantae [39]. A small group of “thermogenic” plants are able to heat their reproductive tissues to temperatures well above ambient by maintaining a very high rate of uncoupled and hence heat-releasing AOX respiration [40–42]. This can function to attract pollinators or provide optimal temperatures for floral development. However, most plants and tissues are inherently non-thermogenic, in which case the presence of AOX must be for other purposes.

While AOX is found throughout the plant kingdom, it is also found, though more sporadically, in the other kingdoms. In extant prokaryotes, AOX is limited to some α-proteobacteria (e.g., *Novosphingobium aromaticivorans*, [43]) and it is likely that AOX entered eukaryotic lineages via the ancient proteobacterial endosymbiont that gave rise to mitochondria [39]. AOX is also found sporadically in protists and fungi, in many cases within pathogenic organisms within these groups [44,45], but also within important non-pathogenic model systems such as the fungi *Neurospora crassa*[46] and *Podospora anserina*[47] Surprisingly, AOX is also present in many animal phyla, although clearly absent from vertebrates and arthropods [39,48]. The finding of AOX in the animal kingdom has spawned several recent studies in which the protein has been introduced into different vertebrates, as a means to study aspects of mitochondrial bioenergetics and signaling [49–51].

### 2.2. AOX Capacity and Activity

The plant AOX is an interfacial membrane protein, oriented toward the matrix side of the IMM and coupling the oxidation of ubiquinol to the four-electron reduction of O_2_ to water. As emphasized earlier, AOX is non-proton pumping and since it bypasses proton-pumping complexes III and IV, electron flow to AOX dramatically reduces the energy yield of respiration.

The maximum possible flux of electrons to AOX is often termed *AOX capacity*. Estimation of AOX capacity is analogous to estimation of an enzyme’s maximum activity. In general, the AOX capacity of a tissue or isolated mitochondria can be estimated by the addition of a cyt pathway inhibitor such as cyanide (CN), followed by the addition of an AOX inhibitor such as salicylhydroxamic acid (SHAM) or *n*-propyl gallate. Then, capacity is generally defined as the O_2_ uptake resistant to the cyt pathway inhibitor and sensitive to the AOX inhibitor. This capacity measure is typically reflective of AOX protein abundance. A more detailed discussion of such measures can be found elsewhere [52,53]. Despite the usefulness of AOX capacity estimates, it is worth emphasizing that AOX capacity does not give any indication of the actual flux of electrons to AOX (*i.e.*, *AOX activity*) in the sample prior to the introduction of inhibitor. The actual flux will depend upon the partitioning (sharing) of electrons between AOX and complex III, since both are utilizing the same substrate, ubiquinol. Hence, to fully understand the importance and metabolic role of AOX respiration, we must be able to reliably estimate AOX activity under steady-state physiological conditions (*i.e.*, in the absence of any ETC inhibitors). The means to measure AOX activity is by an oxygen isotope discrimination technique, a noninvasive method based on the observation that AOX and cyt oxidase discriminate to different extents against heavy O_2_ (^18^O^16^O) [54]. The technique has been applied in both gas-phase systems (typically for use with tissue) and aqueous phase systems (typically for use with isolated mitochondria). More recently, the technique has been advanced to be compatible with field sampling [55–57]. Isotope discrimination measures have confirmed that AOX can be a prominent component of plant respiration, such as shown in Millar *et al.*[58]. Nonetheless, there is still a paucity of tissue measurements of AOX activity (at least relative to measures of AOX capacity) and so work in this area remains essential. One shortcoming is that, in photosynthetic tissue, isotope discrimination measures can only be made in the dark. Hence, there is no means for direct measure of AOX activity during photosynthesis. In this case, the use of transgenic and mutant plants with modified AOX capacity provides an alternate means to examine the importance of AOX during photosynthetic metabolism [59–61]. More discussion of the isotope discrimination technique can be found elsewhere [54,62,63].

### 2.3. Genetic Control of AOX Respiration

The plant AOX is encoded by a small gene family, consisting of two distinct subfamilies termed *AOX1* and *AOX2*[64]. Dicotyledons contain members of both subfamilies while monocotyledons contain only *AOX1* genes. Expression of the *AOX1* genes (such as tobacco and *Arabidopsis AOX1a*) is highly responsive to abiotic and biotic stress, as well as dysfunctions in respiratory metabolism [65]. The *AOX2* genes are generally not responsive, or at least much less responsive, to such conditions. Instead, *AOX2* genes show specific developmental and tissue expression, for example being expressed in reproductive tissues and seeds [66–68].

**Figure 2:**
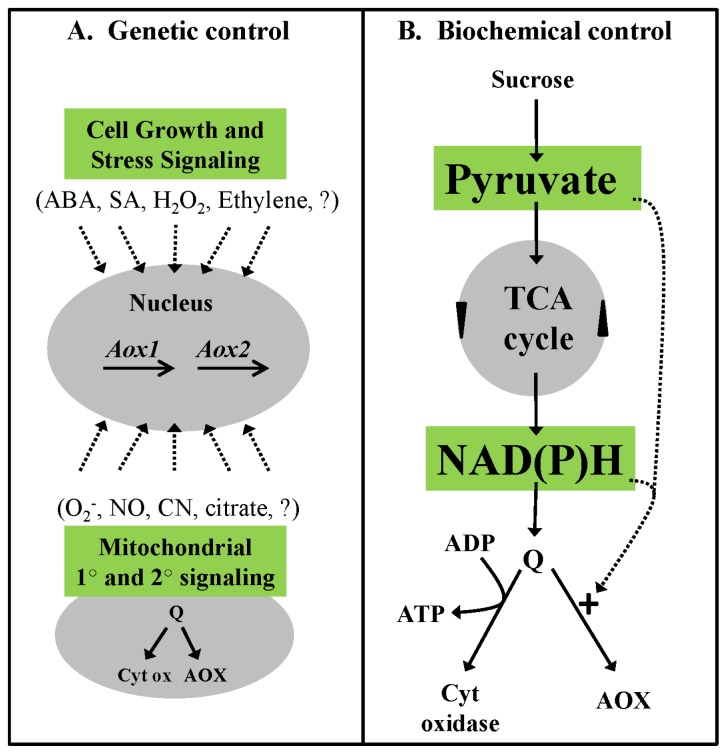
Genetic (**a**) and biochemical (**b**) control of AOX respiration in plants. Genetic control of AOX gene expression likely involves both retrograde signals from the mitochondrion and other cellular signals related to growth, energy metabolism and stress. Biochemical control of the partitioning of electrons to AOX is, at least in part, the result of a feed-forward activation of AOX by upstream carbon (pyruvate) and redox (NAD(P)H) status.

Specific AOX gene family members are strongly induced at the transcript and protein level by complex III or complex IV dysfunction [69,70], suggesting that AOX expression is highly responsive to insufficient cyt pathway capacity downstream of the ubiquinone pool. However, AOX is also commonly induced by complex I dysfunction and by other disruptions in respiratory metabolism such as inhibition of ATP synthase, uncoupling of the ETC, and inhibition of the TCA cycle [71,72]. On the other hand, some studies have reported no change in AOX amount in response to dramatic changes in the ETC [36]. Overall, the results indicate that AOX expression is likely responsive to multiple and complex signals of respiratory status, and effort is being made to elucidate the primary retrograde signals and molecular components able to relay this status to the nucleus to control AOX gene expression (Figure 2).

To identify molecular components of the retrograde pathway(s) controlling AOX expression, studies have exploited the ability of the inhibitor AA to strongly induce AOX [73,74]. A screen for mutants impaired in AOX induction by AA recently identified the nuclear-localized cyclin-dependent kinase E1 as one such regulatory component [74]. Interestingly, this kinase is likely embedded in pathway(s) that modulate plant growth in response to energy and stress signals. Further, functional characterization of the promoter of *Arabidopsis AOX1a* identified a repressor element that was shown to bind the transcription factor ABSCISIC ACID INSENSITIVE 4 (ABI4) [75]. These findings provide a molecular link between AOX expression and signaling by the stress hormone abscisic acid. Interestingly, ABSCISIC ACID INSENSTIVE 4 is also a molecular component of chloroplast retrograde signaling [76], suggesting coordination between organelle retrograde pathways. It was recently shown that over-expression of the *Arabidopsis* WRKY15 transcription factor inhibited the salt stress-induced expression of AOX and other typically stress-responsive mitochondrial proteins [77]. This suggests that WRKY15 may also act to repress AOX expression and is in line with an earlier study identifying W-box motifs within the *AOX1a* promoter [78] (Figure 2).

There is evidence that accumulation of the TCA cycle intermediate citrate may be an important signal controlling AOX expression. In tobacco cells, changes in AOX expression correlated strongly with changes in citrate level induced by disparate means including inhibition of aconitase, acetate feeding, and H_2_O_2_ treatment [71]. However, inhibition of the cyt pathway downstream of ubiquinone, while strongly inducing AOX, did not increase citrate level. Hence, AOX can respond to both the status of the cyt branch of the ETC and to the status of upstream respiratory metabolism, but may use different signaling pathways to accomplish this. Other studies have also shown that citrate is an important signal for AOX expression [35,79]. For example, recent evidence suggests that, under hypoxia, the generation of NO inhibits aconitase, resulting in accumulation of citrate and increased AOX expression [35] (Figure 2).

It is well established that exogenous ROS can induce AOX expression [71,80] and that AOX expression can be attenuated by artificial ROS-scavengers [81]. This suggests that ROS may be an important signal controlling AOX expression. There is compelling recent evidence that specifically O_2_^−^ in the mitochondrial matrix may be a key signal. It was shown that overexpression of MnSOD could lower O_2_^−^ levels in rice mitochondrial matrix, monitored with the mitochondrion-specific O_2_^−^ indicator Mito-SOX [82]. This in turn attenuated the induction of AOX expression by abiotic stresses such as drought, cold and salinity. This attenuation of AOX expression was not seen in plants overexpressing non-mitochondrial SOD isozymes. Interestingly, a couple of studies have shown that bongkrekic acid, a compound known (at least in animals) to inhibit the mitochondrial permeability transition pore is able to block the induction of AOX expression by stress [81,83]. This suggests that pore opening, which may be promoted by ROS [84,85], is an important event in retrograde signaling for AOX induction (Figure 2).

Sulfide, CN and NO are all potent inhibitors of complex IV, and each of these are likely produced within mitochondria, as intermediates in various pathways. While it has been shown that exogenous addition of these compounds to plant tissue will induce AOX expression, the *in vivo* significance of this is not well understood. Effective metabolism or scavenging of these compounds might prevent them inhibiting complex IV, at least under normal physiological conditions.

The plant hormone salicylic acid (SA) has dramatic effects on AOX transcript and protein abundance. In this case, increased transcript abundance may primarily be the result of post-transcriptional mechanisms, as reported for both *Arabidopsis*[80] and the thermogenic species *Sauromatum guttatum*[86]. It is also possible that SA has opposing effects on AOX expression dependent on its concentration. It was reported that low concentrations of SA were effective at inducing AOX transcript and protein but not high concentrations [87] (Figure 2).

Physiological, biochemical and genetic studies suggest that, at least in some cases, AOX may be co-expressed with members of the non-energy conserving alternate dehydrogenases [80,88–91]. The coordinated expression of these components implies that they might function together, representing an ETC path completely uncoupled from ATP generation.

### 2.4. Biochemical Control of AOX Respiration

AOX is member of a non-heme diiron carboxylate family of proteins that are distinguished by an iron-binding motif consisting of six conserved amino acids (four glutamate and two histidine) that coordinate two irons within a structurally conserved four-helix-bundle conformation [92]. Site-directed mutagenesis studies have confirmed the essential nature of these amino acids as well as identifying other residues (e.g., tyrosine residues) essential for activity [93,94]. EPR and FTIR spectroscopy studies have shown that the active site for the reduction of oxygen to water does indeed comprise a binuclear iron center [95,96] and other work has identified residues important for ubiquinol binding [94,97]. No three dimensional structure of any membrane-bound diiron carboxylate protein is yet known but there has been a preliminary analysis of crystals of a trypanosome AOX [98].

Notably, the plant AOX exists in the inner mitochondrial membrane as a homodimer, the state of which dramatically impacts activity of the enzyme. The dimer may be either non-covalently linked (reduced *active* form) or covalently linked by a regulatory disulfide bond between the two monomers (oxidized *inactive* form) [99]. A conserved cysteine residue toward the *N*-terminus and exposed within the matrix (termed Cys I) is responsible for this disulfide bond [100,101]. Reduction of the disulfide bond is facilitated by the oxidation of specific TCA cycle substrates and, based upon the substrate specificity, it is hypothesized that specifically NADPH provides the reducing power for this regulatory reduction [102]. This is in keeping with *in organello* studies showing that a mitochondrial-localized thioredoxin is able to reduce this disulfide bond [103].

Once in reduced form, AOX is sensitive to activation by specific α-keto acids, most notably pyruvate, but also others [102,104,105]. While it is clear that the exposed sulfhydryls are an important site of interaction for pyruvate [100,101], the details of this interaction remain elusive. It has been shown that substitution of Cys I by a charged amino acid (positive or negative) provides increased basal activity, suggesting that a charge-induced conformational change is important [106]. Also, recent studies indicate that the activating effect of pyruvate is due to an effect on the enzyme’s apparent *V*_max_, resulting from the ability of pyruvate to stabilize the active AOX [105]. In some plant AOX isoforms the regulatory Cys I is replaced by Ser and this change confers on AOX the ability to be activated by succinate rather than by α-keto acids [107,108]. The mechanism of this activation is poorly understood. Also, a second conserved and *N*-terminal Cys residue (Cys II), along with other postulated sites may also be important in the activation of at least some AOX isoforms [109,110].

In summary, at least some AOX isoforms (including tobacco and Arabidopsis AOX1a) are subject to sophisticated biochemical control in which electron flow to AOX is activated in a feed-forward manner by upstream respiratory metabolism (Figure 2). This involves a two-step biochemical activation of the AOX protein, a covalent modification followed by an allosteric activation, in response to the redox (NADPH) and carbon (pyruvate) status of the mitochondrial matrix, respectively. Using the isotope discrimination technique, it was shown that plants overexpressing AOX (and thus having higher AOX capacity) did not necessarily show increased AOX activity, highlighting the prominence of the AOX biochemical controls over protein amount in determining the partitioning of electrons between AOX and the cyt pathway [111]. This is in keeping with numerous isotope discrimination studies showing that protein amount is not necessarily a good predictor of AOX activity in different tissues or growth conditions.

### 2.5. Control of Mitochondrial ROS and RNS Generation by AOX

The rate of ROS generation by mitochondria depends upon the reduction state of ETC components. In animals, this reduction state is generally dependent upon the rate of electron transport and the membrane potential, which in turn are primarily dependent upon the rate of dissipation of membrane potential, particularly by oxidative phosphorylation. Hence, when ADP is readily available and being actively phosphorylated to ATP, dissipation of the proton gradient lowers membrane potential and O_2_^−^ generation is less than when ADP is limiting. In plants, however, the relationship between electron transport, oxidative phosphorylation and ROS generation is more complex because electron flow from ubiquinol to AOX does not contribute to membrane potential. Hence, AOX could provide a means to maintain significant electron flow, even when ADP is limiting, while still preventing the over-reduction of the ETC [10,112].

Recent studies have now provided direct *in planta* evidence that AOX acts to prevent the over-reduction of ETC components that leads to single electron leak. Using fluorescent confocal microscopy, it was shown that tobacco leaves with suppressed levels of AOX, due to RNA interference, have increased concentrations of mitochondrial-localized O_2_^−^[113]. The study also found higher levels of NO in the leaves lacking AOX, a portion of which also localized to mitochondria. Since mitochondria have been proposed to generate NO via single electron leak from the ETC to nitrite [13,14], the results with tobacco leaf suggest that AOX, by controlling the reduction state of the ETC, also dampens NO generation. The conclusions of this study are further supported by experiments with AA that, by restricting electron flow, causes an over-reduction of ETC components. In wild-type (WT) plants, both mitochondrial O_2_^−^ and NO increased dramatically in response to AA. However, these increases were not seen in plants overexpressing AOX and hence able to maintain high rates of electron flow, even in the complete absence of complex III activity [114].

## 3. A Role for Alternative Oxidase to Maintain Metabolic Homeostasis

The presence of AOX provides the respiratory system with built-in flexibility regarding the degree of coupling between carbon metabolism pathways, ETC activity, and ATP turnover. Adjustment of the absolute and relative rates of electron flow between AOX and cyt oxidase provides a means to rapidly align cellular demands for ATP with cellular demand for pyridine nucleotide turnover and carbon intermediate supply for biosynthesis. In general terms, this could provide an important mechanism to switch between catabolic and anabolic modes of respiration, as well as providing a general means to control the carbon, energy and redox status of the cell. Below are a few generalized examples to illustrate the potential for AOX to provide such metabolic homeostasis to plant metabolism.

### 3.1. Homeostasis of Carbon Pools—An Example

**Figure 3:**
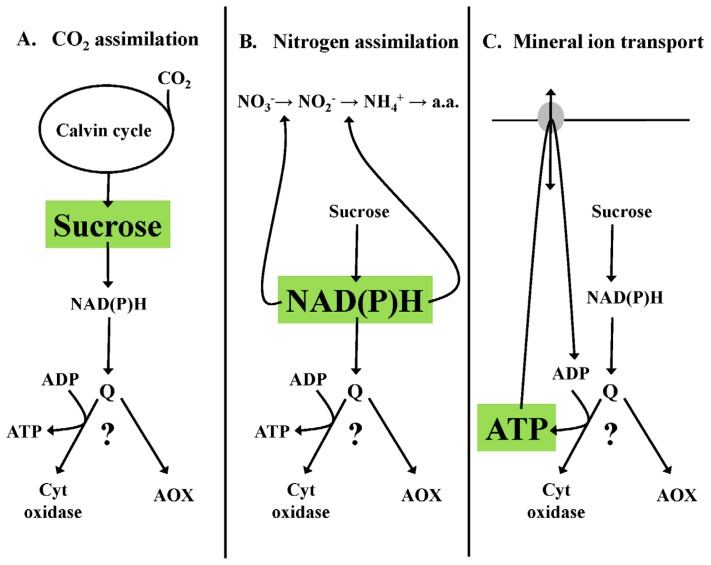
Examples to illustrate how changes in the partitioning of respiratory electron flow between cyt oxidase and AOX can act to maintain metabolic homeostasis. This could include homeostasis of the (**a**) carbon status, (**b**) redox status and (**c**) energy status of the plant cell. See the text (Section 3) for discussion of these examples.

As autotrophic organisms, the carbon status of plants is dependent upon the assimilation of atmospheric CO_2_ during photosynthesis (Figure 3). Besides providing the substrate for growth and maintenance of the plant, carbon status is also an important regulator of photosynthetic capacity and activity. For example, a shift of plants from normal to high CO_2_ can dramatically stimulate photosynthetic activity. However, this increased activity is often curtailed as carbon accumulation is thought to act in a feedback fashion to down-regulate photosynthetic capacity and activity [115]. What is poorly understood is the degree to which increased respiratory metabolism could act to consume the extra carbon assimilated at high CO_2_. For example, AOX activity could provide a means for respiration to consume carbon without this process being impeded by the rate of ATP turnover (Figure 3). How the respiratory system will respond could be of critical importance in defining how photosynthesis will respond to future conditions of higher atmospheric CO_2._ Some reports have begun to examine this potential role for AOX [116,117]. However, this is clearly an area requiring further study.

### 3.2. Homeostasis of Redox State—An Example

The assimilation of nitrogen (N) to amino acids provides a compelling case where variable AOX activity could be important to control the reduction state of the pyridine nucleotide pool (Figure 3). Nitrate assimilation to amino acids has a high demand for reducing power (to reduce nitrate to ammonium) but ammonium assimilation to amino acids has a low demand for such reducing power. Hence, one might expect AOX respiration to be more prevalent in plants grown on ammonium, when there is a greater potential for excess reducing power (Figure 3). Evidence supporting this is the observation that *Arabidopsis* plants grown on ammonium have a higher AOX capacity and higher overall respiration rate than plants grown on nitrate [118]. However, *Arabidopsis* plants lacking AOX also display the higher respiration rate typical of growth on ammonium and it was suggested that this respiration actually occurs primarily by the cyt pathway [119]. Hence, the role of AOX during growth on nitrate *versus* ammonium requires further study to include different plant species, further analysis of plants with altered AOX level, and isotope discrimination measures of AOX activity during growth on nitrate or ammonium.

### 3.3. Homeostasis of Energy Status—An Example

The active transport of mineral ions into the plant is an energy-intensive process supported by plant respiration (Figure 3). As such, plant respiration is often divided into that supporting growth processes, that supporting maintenance processes, and that supporting specifically ion transport processes [120]. Ion transport processes are both dynamic and variable. Hence, the ATP demand for this process is also likely dynamic and variable [121–124]. It is just such variable demand for large amounts of ATP that could necessitate a respiratory system with variable coupling to ATP synthesis (Figure 3). To my knowledge, the use of isotope discrimination to measure root AOX activity in response to variable and defined ion transport scenarios has not yet been reported.

## 4. A Role for Alternative Oxidase to Maintain Signaling Homeostasis

In addition to their primary metabolic function, mitochondria are being increasingly recognized as a “signaling organelle” able to influence cellular processes such as nuclear gene expression and programmed cell death [125,126]. For example, signaling pathways from mitochondria are thought to relay the functional status of mitochondria to the nucleus, in order to coordinate that functional status with the expression of nuclear genes encoding mitochondrial proteins (*i.e.*, retrograde signaling) [17]. ROS, RNS and other metabolites have been implicated as signaling molecules. These potential signaling molecules are closely associated with mitochondrial metabolism, hence providing an important connection between metabolism and signaling events. As outlined below, AOX may be ideally suited to establishing this link between metabolism and signaling.

A key contrast may exist between the AOX role to maintain metabolic homeostasis and the AOX role to maintain signaling homeostasis. Unlike metabolic pathways, signaling pathways are often associated with an amplification of the signal by second messengers. Hence, the changes in AOX activity needed to provide signaling homeostasis may be subtle compared to the changes in AOX activity that provide metabolic homeostasis. For example, assuming ETC-generated ROS as an important signal, it has been shown that small changes in electron transport rate or in membrane potential can have a relatively large impact on ROS generation [127].

### 4.1. Homeostasis of ROS Signaling—An Example

**Figure 4:**
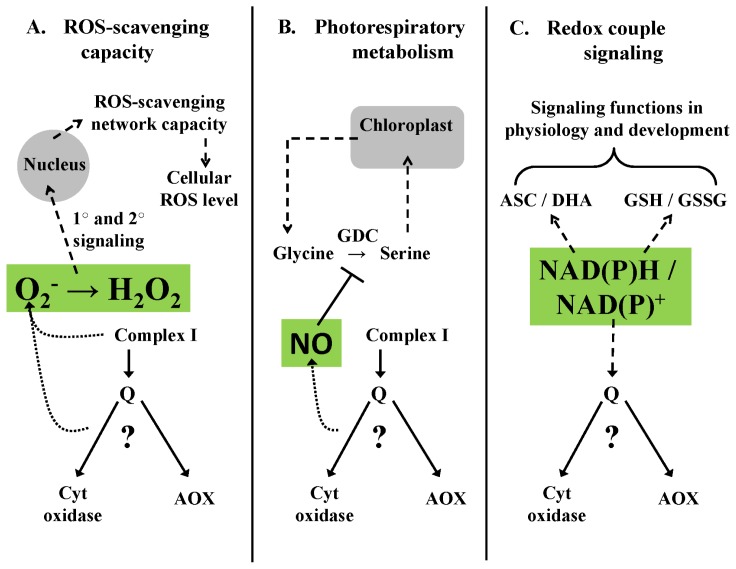
Examples to illustrate how changes in the partitioning of respiratory electron flow between cyt oxidase and AOX can act to maintain signaling homeostasis. This could include homeostasis of (**a**) ROS level, (**b**) NO level and (**c**) the reduction state of different cellular redox couples. See the text (Section 4) for discussion of these examples. ASC, ascorbate; DHA, dehydroascorbate; GSH, glutathione; GSSG, glutathione disulfide.

As outlined earlier, there is now substantial evidence to suggest that AOX has an important influence on ROS generation by the respiratory chain. AOX activity dampens the generation of O_2_^−^, which in turn will reduce its conversion to other ROS species such as H_2_O_2_ and hydroxyl radical (Figure 4). An important implication of this has already been described in transgenic or mutant plants with altered AOX expression. In both *Arabidopsis* and tobacco, lack of AOX has been shown to heighten the expression of ROS-scavenging enzymes throughout the cell [128,129]. This implies that changes in ROS generation by the mitochondrion alone can signal a dramatic change in the ROS-scavenging capacity of the plant cell (Figure 4). In this case, mechanisms controlling mitochondrial ROS generation such as AOX could be of central importance in determining how the cell will manage its ROS load and define its steady-state ROS level.

### 4.2. Homeostasis of RNS Signaling—An Example

As outlined earlier, recent study suggests that AOX can dampen the generation of NO by an over-reduced ETC, in a manner similar to controlling O_2_^−^ generation. An example of the possible importance of controlling NO generation in the mitochondrion relates to photosynthesis and photorespiration (Figure 4). Glycine decarboxylase (GDC) is a matrix-localized enzyme responsible for the conversion of glycine to serine, an important step in the photorespiratory cycle. Reduced GDC activity can impede both photorespiration and photosynthesis [130,131]. Importantly, there is evidence that GDC activity is controlled by mitochondrial NO via *S*-nitrosylation [132]. Hence, control of mitochondrial NO by AOX provides a potential link between AOX and photorespiratory/photosynthetic metabolism (Figure 4). GDC activity is also sensitive to oxidative stress [133], suggesting that both ROS and RNS homeostasis in the mitochondrion may be critical during photorespiration.

### 4.3. Homeostasis of Metabolite Signaling—An Example

The relative partitioning of electrons between the cyt pathway and AOX could impact a number of closely linked mitochondrial metabolites and related parameters including ATP/ADP ratio, membrane potential, pH gradient across the IMM, and matrix concentration of TCA cycle intermediates. Another example is the NAD(P)H/NAD(P)^+^ redox couple, which is then also tightly linked to the key redox couples for glutathione and ascorbate (Figure 4). Each of these redox couples is suggested to have signaling roles in the plant cell [134]. For example, it was shown that modified expression of an external alternate dehydrogenase (that could function in tandem with AOX) impacted stem NADPH/NADP^+^ ratio and that this had a specific developmental effect, suggestive of a signaling role [135] (Figure 4). Interestingly, plants contain homologs of sirtuin proteins. In animals, mitochondrial sirtuins can catalyze an NAD^+^-dependent de-acetylation of target proteins. This post-translational control of target protein function is thought to act as a signal, linking mitochondrial metabolism (via NAD^+^ level) to events such as changes in gene expression [136]. Interestingly, it was recently shown that numerous plant proteins, including mitochondrial-localized proteins, are acetylated [137,138]. A link may also exist between AOX activity and ascorbate synthesis, which is coupled to the reduction of cyt c in a reaction catalyzed by the IMM enzyme l-galactone-1,4-lactone dehydrogenase [139]. Using transgenic *Arabidopsis* with modified AOX level, it was shown that AOX promoted rates of ascorbic acid synthesis, possibly due to it increasing the availability of oxidized cyt c [140]. On the other hand, tobacco plants lacking AOX were still able to rapidly increase their ascorbate pool following a shift to low temperature [141].

## 5. Alternative Oxidase during Abiotic and Biotic Stress

This section highlights studies that have examined changes in AOX capacity and activity in response to different abiotic and biotic stress.

### 5.1. Temperature Stress

Perhaps the most studied abiotic condition in relation to AOX respiration is temperature, particularly low temperature. Studies in many species have shown a sharp increase in AOX transcript and/or protein after transfer to or growth at low temperature [133,141–152]. In a callus culture of *Arabidopsis*, evidence indicates that ethylene is required for the induction of AOX capacity following a shift to chilling temperature [153]. In tomato, it was reported that a chilling-tolerant genotype showed strong up-regulation of AOX in comparison to a chilling-sensitive genotype [154].

Using isotope discrimination, changes in AOX activity have been determined in response to instantaneous (*i.e.*, minutes to hours), short-term (several hours to several days) and long-term (weeks to months) changes in temperature. In several species, instantaneous changes in temperature were found to have little impact on the relative partitioning of electrons between the cyt pathway and AOX over a wide temperature range [155]. However, in chilling-sensitive maize, a short-term cold treatment (5 days at 5 °C) was associated with a dramatic decline in cyt pathway activity and increase in AOX activity, such that approximately 60% of total respiration was occurring through AOX [156]. Similarly, in *Arabidopsis*, there was a transient increase in AOX activity with sustained chilling (over first 10 days at 5 °C), followed by a return to lower activity in the longer-term [148]. In this case, the transient increase in activity did not require any increase in AOX protein. Alternatively, Fiorani *et al.*[145] found that *Arabidopsis* grown at 12 °C had higher AOX protein amount than warm grown plants and that growth at the low temperature was compromised in knockdown plants with reduced AOX expression, suggesting an important longer-term role for the AOX pathway. Also, Searle *et al.*[157] found that field-grown alpine grasses had higher AOX protein levels (relative to cyt pathway) in the cold months and other studies have also found higher AOX protein in plants grown under low temperature [147,152]. It seems possible that the response of AOX to temperature will be species and tissue-dependent, dependent upon the severity and length of the cold treatment, and dependent upon the developmental or physiological status of the plant at the time of cold treatment. Results, however, do support that AOX respiration commonly becomes more prevalent during both sustained short-term chilling and longer-term growth at low temperature.

Accumulation of soluble carbohydrate is a common response of plants to low temperature. Since one role of AOX could be to manage excess carbohydrate, without this being constrained by rates of ATP turnover, studies have examined the impact of AOX expression on cold-induced carbohydrate accumulation. In *Arabidopsis*, plants lacking AOX did indeed accumulate more starch and displayed a higher carbon to nitrogen ratio than WT upon a shift to low temperature [151]. However, in tobacco, transgenic plants lacking AOX actually accumulated less carbohydrate than WT upon shift to cold, while plants overexpressing AOX accumulated more [141]. This result suggests that changes in AOX activity at lower temperature are likely not being driven simply by changes in carbohydrate availability for respiration.

Upon shift to cold, tobacco plants exhibited a strong increase in AOX transcript and protein and, as expected, a rapid increase in the transcript level of ROS-scavenging enzymes found throughout the cell. There is some data to suggest that AOX amount influences the rapid acclimation responses of tobacco to lower temperature. Upon shift to cold, transgenic plants with strong suppression of AOX showed further enhanced accumulation of transcripts encoding ROS-scavenging enzymes, compared to WT [141]. As a result, these plants actually showed a decline in oxidative damage (lipid peroxidation) after transfer to cold, compared to the slight increase seen in WT plants. One interpretation is that the lack of AOX enhanced a mitochondrial stress-signaling pathway that is able to control the ROS-scavenging capacity of the cell. In this case, the strong induction of AOX in WT plants in response to cold may not be so much as to dramatically increase AOX respiration rate, but rather to increase it just enough to modulate activity of the stress-signaling pathway.

Isotope discrimination studies have also provided some evidence that AOX respiration becomes a more prevalent component of total respiration at high temperature extremes. Experiments involving both instantaneous changes in temperature [111] and longer-term changes in temperature [158–160] provide support for this. Further, rice seedlings with constitutive over-expression of AOX were reported to be more growth tolerant at high temperature than WT rice [161] and comparison of a heat-sensitive and heat-tolerant *Agrostis* species showed that, in the tolerant species, growth at high soil temperature was associated with an increased proportion of AOX respiration [158].

### 5.2. Drought Stress

During drought stress, respiration becomes a more important component of overall plant carbon balance since photosynthetic rate is typically strongly impacted by drought, due to both stomatal and biochemical limitations, while respiratory rates are much less impacted [162–164]. As such, one might expect considerable acclimation of respiratory metabolism during drought. Nonetheless, surprisingly few studies have examined specifically the role of AOX during drought stress. An increase in AOX protein or capacity in response to drought has been noted in wheat leaves [165,166], while no increase in AOX protein was evident in soybean leaves [167] and drought was reported to decrease leaf AOX transcript in *Medicago*[168]. Despite these differences, however, several studies do indicate that AOX respiration is of functional significance under drought. Using isotope discrimination, Ribas-Carbo *et al.*[167] showed that, in soybean leaves, AOX was responsible for near 40% of total O_2_ consumption, compared to approximately 10% in well-watered plants. Drought stress also increased AOX activity as a percentage of total electron flow in *Nicotiana sylvestris*[169]. In wheat, inhibition of AOX (using SHAM) resulted in reduced photosynthetic performance under drought, particularly under high light conditions [165], although such inhibitor-based experiments should be evaluated with caution. An *Arabidopsis* AOX knockout has also been reported to have impacts on photosynthesis and again, this effect was particularly evident under a stress combination that included drought [129]. Based on studies to date, it seems possible that AOX could be of considerable significance during drought stress, particularly to support metabolism in the light, but further study in this area is required.

### 5.3. Nutrient Limitation

Phosphate (P) deficiency has been shown to increase AOX capacity in a number of plant species and tissues including bean root [170], leaves of bean and *Gliricidia sepium*[171], tobacco suspension cells [172] and *Arabidopsis* seedlings [173]. Further, leaf isotope discrimination measurements have shown increased AOX activity in at least some plant species when they lack adequate P [171].

A common metabolic consequence of P deficiency is a significant reduction in the concentration of adenylates and P_i_. Since ADP and P_i_ are critical substrates for oxidative phosphorylation, it was suggested that induction of AOX could represent a means to maintain carbon metabolism and electron flow during P deficiency, when these substrates are in short supply [174,175]. To examine this, WT tobacco cells have been compared to transgenic cells lacking AOX. Under P limitation, cells lacking AOX did indeed show a restricted rate of respiration, as well as changes in carbon and nitrogen metabolism, accumulation of carbohydrates, and an increase in cellular ROS level [172,176]. Using the same system, it was shown that AOX respiration was also important to dampen ROS generation during the high rate of respiration that supported rapid P uptake when it was resupplied to cells [177].

AOX capacity has also been shown to increase during N deficiency in both spinach leaf [178] and tobacco cells [176], while relief of N limitation in the field was shown to reduce the percentage of total electron flow being partitioned to AOX [179]. Transgenic tobacco cells lacking AOX accumulated carbohydrate under N deficiency, similar to that observed during P deficiency. However, unlike the case with P deficiency, the lack of AOX during N deficiency did not increase the expression of several ROS-responsive genes. This suggests that, while AOX had an important role in dampening ROS generation during P limitation, this was not the case during N limitation. This difference may relate to the fact that, while P limitation likely restricts the availability of ADP and P_i_, this should not necessarily be the case during N limitation. A restricted availability of ADP and P_i_ necessitates the need for AOX in order to prevent over-reduction of the ETC and the concomitant ROS generation [176].

### 5.4. Salt Stress

Studies suggest that salt stress negatively impacts mitochondria, resulting in decreased electron transport activities, increased mitochondrial ROS and lipid peroxidation, and the induction of mitochondrial ROS-scavenging systems [180–183]. In tobacco, it was shown that short-term salt stress resulted in a strong elevation of leaf O_2_^−^, with only a more moderate increase in H_2_O_2_[184].

The induction of AOX transcript and protein by salt stress has been shown in a number of plant species including *Arabidopsis*[185,186], pea [187], tobacco [184] and poplar [188]. In a callus culture of *Arabidopsis*, evidence supports a role for ethylene in the induction of AOX capacity by salt stress [189]. In pea, isotope discrimination experiments demonstrated that long-term (14 d) salt stress strongly decreased leaf cyt pathway respiration, while AOX respiration was maintained and now accounted for near 50% of total electron flow [187]. These results suggest a major role for AOX in the respiratory activity of salt stressed pea leaves.

### 5.5. Ozone

AOX respiration may be expected to have a prominent role under stress conditions that strongly impair the cyt pathway. A good example of this may be acute ozone exposure. A 5 h ozone exposure was shown to half the cyt pathway capacity in tobacco leaf [190]. The loss of cyt pathway may have been due to the demonstrated partial loss of cyt c from matrix to cytosol. A transient increase of NO was also seen, which may have inhibited cyt oxidase. The loss of cyt pathway was accompanied by an increased AOX expression and capacity. The increased AOX expression was dependent upon both the increase in NO [190] and a protein phosphorylation event [191]. The ability of acute ozone exposure to increase AOX expression has also been shown in *Arabidopsis*[192].

### 5.6. Metal Toxicity

Many ETC and matrix proteins could be susceptible to metal-catalyzed or metal-enhanced oxidative reactions that impact their function [193,194]. Aluminum (Al) can be an important factor limiting the growth of plants in acidic soils. Al has been shown to inhibit respiratory oxygen consumption in roots, leaf protoplasts, suspension cells and isolated mitochondria from several species [195–197]. In leaf protoplasts of *Arabidopsis*, this inhibition was shown to be accompanied by an increase in matrix O_2_^−^ (measured using Mito-SOX), strongly suggesting that Al can cause ETC dysfunction and increase electron leak from the chain to O_2_[196]. This was corroborated by analyses of mitochondria isolated from Al-treated protoplasts, which showed a time and Al concentration-dependent loss of maximal complex I and complex III activities. This was accompanied by a decline in Fe-S protein, suggesting that Al inhibition was perhaps due to its interaction with the Fe-S centres of the respiratory complexes. In some cases, Al treatment has been shown to increase the capacity of AOX respiration, perhaps a response by the cell to ETC dysfunction [196]. It is likely that other metals known to interfere with cyt pathway function (such as cadmium) may also induce AOX respiration, as shown in the protist *Euglena*[198]. Finally, excess copper has also been shown to inhibit respiration and induce the level of AOX transcript and protein [199].

### 5.7. Low Oxygen

AOX amount (transcript, protein or capacity) has been reported to change in response to hypoxia, anoxia, or re-oxygenation after a low oxygen treatment. This has been examined in leaves and roots of several species [200–207]. No clear pattern has yet emerged from these studies. For example, AOX has been shown to either increase or decrease in response to hypoxia. Hence the importance of AOX for acclimation to such stresses remains unclear. AOX has a lower affinity for O_2_ than cyt oxidase [208,209], making it seem less likely that it would have a prominent activity under low oxygen conditions. However, perhaps it is just such lack of activity that could act as a signal for hypoxic conditions. Further study using plants with altered AOX expression might be a useful approach to examine this further. It also remains possible that AOX has a role during re-oxygenation, as suggested earlier [203].

### 5.8. Bacterial Pathogens

SA, ROS (particularly H_2_O_2_ and O_2_^−^) and RNS (particularly NO and peroxynitrite) are thought to be important signaling molecules to initiate and coordinate plant defense responses, such as the HR, during incompatible interactions with bacterial pathogens [210–212]. Strong links also exist between these signaling molecules and mitochondria. For example, SA can disrupt mitochondrial function in disparate ways, depending upon its concentration [213] and NO is a potent inhibitor of cyt oxidase, but not AOX [214]. Further, exogenous additions of SA, H_2_O_2_ or NO have been shown to increase AOX amount in many species and tissues [71,80,190], an effect that likely stems, at least in part, from the ability of these compounds to alter mitochondrial function. AOX expression also responds strongly to bacterial infection [30,215–218]. However, it has been difficult to establish the relationship between changes in AOX amount and activity, the type of bacterial interaction (compatible or incompatible) and downstream responses, such as the HR or changes in defense gene expression.

Recent studies examined the role of AOX during interaction of *Nicotiana tabacum* with *Pseudomonas syringae*. It was shown that the incompatible *P. syringae* pv. *maculicola* induced defense responses that included the HR, and that the HR was preceded by an early and persistent increase of O_2_^−^ in the mitochondrial matrix, which was monitored using Mito-SOX [87]. The “O_2_^−^ burst” was specific to the HR-inducing incompatible interaction, not being seen in response to a compatible (*i.e.*, disease-causing) pv. or in response to the incompatible pv. *phaseolicola* that induced well-known defense responses but not including the HR. The disparate effect of the two incompatible pv.’s appeared to be due to a coordinated response of AOX (as a means to modulate the rate of O_2_^−^*generation*) and MnSOD (the sole enzymatic means to *scavenge* matrix O_2_^−^). While pv. *phaseolicola* infection resulted in a strong induction of AOX and a maintenance of high MnSOD activity, pv. *maculicola* infection failed to induce AOX and was accompanied by a loss of MnSOD activity [87]. Additional studies showed that, in transgenic AOX knockdown plants unable to induce AOX in response to pv. *phaseolicola*, a O_2_^−^ burst was now generated in response to infection [114]. Also, knockdown plants infected with pv. *maculicola* displayed a delayed O_2_^−^ burst that manifested itself in a delayed HR. Overall, the results place AOX as a potential key regulator of a mitochondrial O_2_^−^-based signaling pathway that subsequently impacts plant responses to bacterial infection.

### 5.9. Viral Pathogens

A large number of studies have examined AOX amount (in both local and systemic leaves) in response to viral infection. Early studies established the presence of a SHAM-sensitive pathway of viral resistance, hence implicating a central role for AOX [219]. However, experiments with tobacco AOX knockdown and AOX overexpression plants do not support a critical role of AOX in viral resistance [220,221]. In the transgenic plants, neither N-gene mediated or SA-induced resistance, nor systemic acquired resistance (SAR) was significantly altered. Nonetheless, studies to date do suggest that the mitochondrion in general (and perhaps AOX in particular) as having a role in the virus-induced HR. For example, smaller N-gene mediated HR lesions are seen in tobacco plants overexpressing AOX [220] as well as in tobacco plants that lack complex I (CMSII plants) and show a constitutive increased amount of AOX [222]. Recently, immunogold labeling was used to examine compartment-specific changes of glutathione in tobacco responding to an incompatible (*i.e.*, HR-inducing) infection with tobacco mosaic virus (TMV) [223]. Interestingly, the mitochondrion exhibited a decline in glutathione level in response to TMV while its level tended to increase (or show little change) in the five other compartments examined. This study further emphasizes the potential importance of mitochondria during the HR defense response to virus.

Several recent studies have implicated the importance of AOX in the establishment of SAR (e.g., [224,225]) but this interpretation is largely dependent upon experiments that involve long-term incubation of plant tissue with SHAM and other respiratory chain inhibitors, an approach that has been criticized such as by Ordog *et al.*[220].

### 5.10. Fungal Pathogens

Several studies have shown that respiration and mitochondrial activity are enhanced following infection with plant pathogenic fungi [226–229]. Further, several studies suggest an active role of mitochondria in plant responses to fungi, such as the early generation of mitochondrial ROS shown to precede a fungal elicitor-induced HR [230] or a fungal toxin-induced cell death [231]. Fungal infection is also associated with generation of NO and CN, that could potentially perturb mitochondrial function [232,233]. However, relatively little work has been reported to establish whether AOX has a critical role in plant responses to pathogenic fungi.

## 6. Alternative Oxidase, Plant Growth and Stress Tolerance

This section highlights studies that have shown an effect of AOX respiration on plant performance (e.g., growth, photosynthesis) or plant tolerance to different biotic or abiotic stress.

### 6.1. A Role for AOX in the Optimization of Photosynthetic Metabolism

Photosynthesis and respiration are interdependent processes. This interaction in the light depends upon a direct or indirect exchange of common metabolites between chloroplast and mitochondrion via the cytosol [234–237]. Since photosynthesis has dramatic impacts on the supply and demand of ATP, NAD(P)H, and carbon intermediates, it has been postulated that this will necessitate changes in AOX activity. Numerous lines of evidence do suggest that AOX activity is important for at least the optimization of photosynthesis. Artificial inhibition of AOX has been shown to reduce CO_2_ assimilation and/or oxygen evolution [165,235,238–241]. Further, the expression and protein level of AOX is dependent upon irradiance [242,243], AOX transcript amounts show a diurnal pattern that peaks early in the light period [87,222], and higher irradiance can shift the AOX protein from its oxidized inactive to reduced active form [244]. Further, an AOX in wheat was shown to be most strongly activated at the biochemical level by photorespiratory intermediates, rather than pyruvate [245]. Isotope discrimination experiments have shown that AOX activity in the dark is higher in plants given higher irradiances in the preceding light period [157], and also that “sun” species have higher AOX activity than “shade” species [246]. Also of interest, *Arabidopsis* plants defective in components that contribute to control of the chloroplast redox state have increased AOX amount. These include plants defective in cyclic electron transport [247] or lacking plastid terminal oxidase, a plastoquinol oxidase in the thylakoid membrane that could act as an alternate electron sink in the chloroplast [248]. In these cases, increased AOX may compensate for the function of these chloroplast components in stabilizing chloroplast redox state. Indeed, a common theme of many of the above studies is that AOX activity may optimize photosynthesis by oxidizing “excess” reducing power, when the generation of NADPH in the chloroplast exceeds its use by the Calvin cycle and other anabolic metabolism. Such conditions are likely to be particularly prevalent during abiotic stress [7]. Recently, it was shown that an AOX2 isoform in *Arabidopsis* localizes to thylakoid membranes rather than the mitochondrion, or perhaps that it is dual-targeted to both organelles, revealing a potential unexpectedly close interaction between AOX and photosynthetic electron transport [249].

A role for AOX during photosynthesis has also been investigated in transgenic and mutant *Arabidopsis* plants. Microarray analyses showed that transcripts encoding chloroplast proteins, particularly those associated with the light reactions of photosynthesis and stress are amongst the most perturbed genes in plants lacking AOX [24,129,250]. Several studies suggest that photosynthetic characteristics are perturbed in these plants, particularly during periods of stress [59–61,67,129,251]. The mechanism(s) responsible for these perturbations and the specific means by which AOX interacts with and aids photosynthesis is not yet well understood and represents an important area for further study.

### 6.2. A Role for AOX during Specific Perturbation of the ETC

Many plants store cyanogenic glycosides in their vacuole as a defense against herbivory. If released to the cytosol by mechanical damage, these cyanogens are rapidly degraded to release CN. In the cyanogenic crop plant cassava, harvesting of the tuberous roots has been shown to result in a rapid burst of ROS that strongly curtails post-harvest shelf-life. It was recently shown that this ROS burst is dependent upon CN release, caused by the mechanical damage associated with harvesting [252]. This suggested that the ROS might be mitochondrial in origin, arising due to the inhibition of cyt oxidase by CN. Supporting this idea, over-expression of AOX in the tubers could prevent the ROS burst associated with harvesting and dramatically extended the shelf-life of the tubers [252]. This study is an excellent example of the means by which AOX amount can influence cellular levels of ROS and the downstream events dependent upon that ROS.

In non-cyanogenic plants, the major source of CN in the cell occurs during ethylene biosynthesis. Conversion of 1-aminocyclopropane-1-carboxylic acid (ACC) to ethylene by ACC oxidase generates an equimolar amount of CN. β-cyanoalanine synthase (CAS) can catalyze the conversion of the CN and cysteine to β-cyanoalanine and hydrogen sulfide. Similar to CN, hydrogen sulfide is also a potent inhibitor of cyt oxidase. *O*-acetylserine(thiol)lyase C (OAS-C) can catalyze the conversion of the hydrogen sulfide and *O*-acetylserine back to cysteine. It has recently been shown that such a cycle in the mitochondrion is critical to managing cellular levels of CN and hydrogen sulfide. An Arabidopsis T-DNA mutant lacking the mitochondrial CAS isoform was shown to accumulate CN [253]. Interestingly, the mutant showed a higher AOX capacity and higher total respiration rate than the WT. A possibility is that AOX activity is higher in the mutant (due to partial inhibition of cyt oxidase) and that the lower ATP yield is compensated by increasing the total electron flow to O_2_. Similarly, mutation of the mitochondrial OAS-C isoform resulted in accumulation of hydrogen sulfide and CN, accompanied again by increased AOX and higher total respiration rate [254]. Interestingly, both of the above mutants display a defect in root hair formation, suggesting a link between root hair initiation and the mitochondrion. Since ROS are generally recognized as an important signal for root hair development [255], an intriguing possibility is that changes in a mitochondrial ROS signature due to the changes in electron transport has a role in the root development defect of these mutants. Interestingly, AOX also appears important in protection of plants against bacterial cyanogenesis. When *Arabidopsis* were co-cultivated with CN-producing bacterial strains, plant growth was more strongly curtailed in the transgenic plants lacking AOX [256].

### 6.3. A Role for AOX during Oxidative Stress Conditions

As noted earlier, ozone treatment was found to rapidly reduce cyt pathway capacity and increase AOX capacity in tobacco plants [190]. Hence, another study examined whether constitutive over-expression of AOX would benefit plants subsequently treated with ozone. Paradoxically, it was found that AOX over-expression increased ozone-induced damage. Further examination showed that AOX over-expression slowed the up-regulation of the ROS-scavenging network usually seen in response to ozone treatment, resulting in a more sustained accumulation of ROS in the cell [257]. Such results support the hypothesis that AOX negatively influences a signaling pathway able to increase the ROS-scavenging network of the cell.

As discussed earlier, salt stress has been shown to disrupt mitochondrial function and increase AOX respiration. Recently, constitutive overexpression of AOX was reported to increase the salt tolerance of *Arabidopsis*, improving growth and decreasing shoot sodium level [90]. The AOX-overexpressing plants displayed lower levels of H_2_O_2_ than the WT during stress and the authors suggested that this change may have influenced sodium transport, allowing the plants to better exclude sodium from the shoot [90].

In summary, the ability of AOX overexpression to increase tolerance toward abiotic conditions associated with oxidative stress such as ozone or salt has met with mixed results. On the one hand, increased AOX during the stress may increase tolerance by reducing ROS generation by the respiratory chain, as suggested by the salt stress study. On the other hand, AOX overexpression might interfere with the normal signaling pathways required to engage acclimation responses to stress, as suggested by the ozone study. In this case, while the increased AOX might still dampen ROS generation by the respiratory chain, it could also paradoxically result in an increased total cellular ROS level. The different outcome in these two studies may relate to the different stresses applied (ozone *versus* salt) or may indicate that expression of the ROS-scavenging network in response to AOX manipulation differs between the two species. In tobacco, AOX over-expression perturbed expression of the ROS-scavenging network during the stress [257] but the *Arabidopsis* study did not report on the expression of the ROS-scavenging network [90].

### 6.4. A Role for AOX in Defining Susceptibility to Programmed Cell Death

As discussed earlier, acute Al exposure can inhibit components of the respiratory chain and increase mitochondrial ROS generation [195–197]. In *Arabidopsis* leaf protoplasts, such acute exposure was shown to result in cell death [196]. The cell death was associated with DNA laddering and could be effectively blocked by a ROS scavenger (ascorbate) or by cyclosporine A, suggesting that the death was programmed and involving the mitochondrion. Significantly, *Aox1a* knockdown protoplasts showed increased susceptibility to cell death, indicating that the induction of AOX capacity seen in the WT in response to Al was having a protective role against cell death. Further, cells overexpressing AOX were less susceptible to Al-induced cell death than the WT [196]. Tobacco plants and suspension cells lacking AOX have also been shown to have an altered susceptibility to cell death signaling molecules [128]. In this case, it was shown that susceptibility to SA and NO correlated with the steady-state cellular level of ROS (prior to SA or NO treatment) and that AOX amount contributed to this steady-state in part by influencing expression of the cellular ROS-scavenging network.

### 6.5. A Role for AOX in Protection against Biotic Stress

Recently, transgenic tobacco plants with silenced *Aox1a* expression were used to evaluate the role of AOX in protection against three disparate biotic stresses, a chewing herbivore, a piercing-sucking insect, and a bacterial pathogen [258]. While each of these challenges induced AOX in WT plants, lack of AOX had no impact on resistance against the chewing herbivore. On the other hand, lack of AOX did impact response to the other two challenges. In plants lacking AOX, the piercing-sucking insect caused more leaf damage. Also, HR-like cell death in response to bacterial infection occurred more rapidly in the plants lacking AOX. Interestingly, in both cases, SA levels were higher in the challenged plants lacking AOX than challenged WT plants [258]. In the case of the insect, the high SA appeared to inhibit jasmonic acid-dependent defenses against insect feeding. The higher SA in response to the bacterial infection may have promoted cell death by accelerating mitochondrial dysfunction. This study hints that AOX level may (indirectly?) influence SA level, an important determinant of many biotic stress responses. This relationship deserves further investigation.

### 6.6. The Relation of AOX with Overall Growth and Productivity

The relationship between AOX respiration and overall plant growth/yield remains poorly understood. Theoretically, the presence of AOX in plants could negatively impact growth since its activity reduces the respiratory yield of ATP, an important requirement for growth. However, perhaps the ability of AOX to maintain metabolic and signaling homeostasis, as outlined in this review, can outweigh the energy cost and positively impact growth, particularly under variable (stress) conditions.

The relationship between AOX and growth has been examined in a simple tobacco suspension cell system, and with growth of the cells in macronutrient-sufficient or macronutrient (N or P) deficient conditions [176]. Under macronutrient limitation, there is presumably a large imbalance between the supply and demand of carbohydrate for growth, since demand is being strongly curtailed by the nutrient deficiency. WT cells responded to the macronutrient limitation by inducing large amounts of AOX, which was associated with a reduced carbon use efficiency (efficiency of conversion of medium sugar into biomass), and a large increase in the ratio of respiration rate to growth rate. However, cells unable to induce AOX displayed no change in carbon use efficiency or the ratio of respiration rate to growth rate. As a result, these cultures accumulated much more biomass under nutrient limitation than did the WT cultures. The interpretation is that WT cells under nutrient limitation continued to burn the abundant carbohydrate but uncoupled this from growth by utilizing the non-energy conserving AOX. In the absence of AOX, transgenic cells also burned the abundant carbohydrate but in this case were unable to uncouple this from ATP generation and growth. The inevitable consequence of this was a severe drop in the tissue concentration of the limiting nutrient in transgenic cells compared to WT. This study suggested that, at least in a system with single plant cells bathed in abundant external carbohydrate, AOX was a critical component to fine-tune growth rate in response to nutrient availability [176].

Another single cell system in which the relation between AOX and growth has been critically examined is in the green alga *Chlamydomonas reinhardtii*[259]. Similar to the case with tobacco suspension cells (although not requiring nutrient-limiting growth conditions), transgenic *Chlamydomonas* lacking AOX displayed a large increase in biomass accumulation compared to WT cultures. This was associated with a large increase in cell volume, also similar to that described for tobacco cells under P limitation [172]. The increased biomass accumulation in *Chlamydomonas* was consistent with a proteomic analysis that showed an up-regulation of proteins associated with anabolism and attendant down-regulation of catabolism-related genes [259]. Since catabolism is a net generator of reducing power while anabolism is a net consumer, this acclimation by cells lacking AOX could help avoid over-reduction of the respiratory chain and its associated ROS production.

In general, while the single cell systems discussed above have shown that removing AOX had a positive impact on growth, the relation between AOX and growth will undoubtedly be much more complex in whole plants. A recent example that illustrates this complexity comes from experiments in which AOX was overexpressed in cassava tubers [252]. Under optimal greenhouse conditions, six of seven transgenic lines overexpressing AOX exhibited a significantly higher tuber fresh weight than WT, suggesting that any negative effects of AOX overexpression were being more than offset by other positive effects. However, when three of these seven transgenic lines were grown under variable field conditions, the yield of two lines was dramatically reduced relative to WT. In this example, the factors that may be driving the positive and negative yield changes in greenhouse and field have not yet been reported and represent an important area for future research.

In *Arabidopsis* plants growing at low temperature, AOX was reported to beneficially impact early vegetative growth since antisense knockdown of *Aox1a* slowed early growth while constitutive overexpression accelerated early growth [145]. A beneficial impact of AOX was also noted in another study examining an *Aox1a* T-DNA mutant of *Arabidopsis*. In this case, neither the AOX mutant nor a mutant in cyclic electron transport showed any growth defect at high light. However, the double mutant showed strongly curtailed growth [60]. At present, the mechanism(s) responsible for the growth phenotypes in the above studies remain obscure. However, such findings do strongly suggest that the presence of AOX is beneficial for plant growth (at least under some growth conditions), despite its non-energy conserving nature. This conclusion is consistent with isotope discrimination experiments that found a positive correlation between AOX activity and growth rate across six grass species [260]. However, other isotope discrimination studies have found a negative correlation between AOX activity and growth, such as in non-stressed soybean roots [58] and non-stressed *Arabidopsis* leaf [261]. Recently, Chai *et al.*[262] were the first to report the impact of altered expression of an AOX2 gene family member on growth. In this case, antisense knockdown of an *Aox2b* gene in soybean was shown to compromise both vegetative growth and seed yield under typical greenhouse growth conditions.

In another study comparing growth of *Arabidopsis* plants with altered AOX amount, it was reported that knockdown and overexpression plants displayed similar relative growth rate as WT plants under optimal growth conditions. However, under stress (drought) conditions, plants overexpressing AOX displayed a higher relative growth rate than WT plants [263]. Further, while the plant lines all displayed similar relative growth rate under optimal growth conditions, there were nonetheless differences in plant size in the early stages of growth, with plants lacking AOX being larger and plants overexpressing AOX being smaller than WT. These differences in plant size between lines disappeared when plants became larger. Overall, these data strongly suggest that plant growth is particularly sensitive to AOX amount during seedling establishment and during periods of stress [263]. The above studies suggest that the relationship between AOX respiration and plant growth is indeed complex and that, while AOX respiration is costly in terms of energy conservation, this cost can be outweighed by beneficial roles of this pathway.

## 7. Conclusions

Stress can perturb metabolism, such as by disruption of enzymes and membrane processes. Stress likely similarly disrupts signal transduction pathways, as well as engaging signal pathways associated with stress acclimation. Over the past decade, AOX has emerged as an important mitochondrial component of the plant stress response. Such work emphasizes that numerous abiotic and biotic stresses impact on mitochondrial bioenergetics and function. Stress alters the cellular demands on mitochondrial metabolism and changes in AOX activity represent a means to rapidly meet these changing demands. AOX activity can also directly impact the level of potential important signaling molecules, thus providing an important link between mitochondrial function, signal transduction, and acclimation to stress.

## Abbreviations

AA: antimycin A
ACC: 1-aminocyclopropane-1-carboxylic acid
AOX: alternative oxidase
CAS: β-cyanoalanine synthase
cyt: cytochrome
ETC: electron transport chain
GDC: glycine decarboxylase
G6PDH: glucose-6-phosphate dehydrogenase
HR: hypersensitive response
IMM: inner mitochondrial membrane
IMS: inner membrane space
MnSOD: manganese superoxide dismutase
NO: nitric oxide
O_2_^−^: superoxide
OAS-C: O-acetylserine(thiol)lyase C
OPP pathway: oxidative pentose phosphate pathway
RNS: reactive nitrogen species
ROS: reactive oxygen species
SA: salicylic acid
SAR: systemic acquired resistance
SHAM: salicylhydroxamic acid
TCA cycle: tricarboxylic acid cycle
TMV: tobacco mosaic virus
WT: wild-type
